# Magneto-Electricity in Various Diseases

**Published:** 1844-03

**Authors:** 


*Magneto-Electricity in various Diseases.*—We copy from Dr. Walkly's pamphlet cases of several diseases successfully treated by magneto-electricity.

*Amenorrhœa.*—H., a young lady, aged 13; has never menstruated, but instead is subject to a cutaneous eruption every fourth week. This commenced three years since, and usually continues from four to six days. Has had an epileptic attack, occasioned by the sudden disappearance of the eruption from the use of cold applications, and also a choreal affection of the left side from a similar cause. May 11th, commenced the daily use of electricity, by placing her feet in a vessel of warm water with one conductor and applying the other to the nerves proceeding from the first and second lumbar vertebra. This was four days previous to the usual appearance of the eruption. The fourth application was followed by the menstrual discharge in the place of the habitual cutaneous affection. No pain of the back preceded its appearance. Since then her general health has been good and her periods regular.

*Chorea.*—Miss ——, aged about 14, was attacked with chorea of the right side in August, 1842. It was supposed to have been occasioned by fatigue, consequent upon watching in a sick family, and was of a very annoying character. It was impossible for her to keep the affected side and limbs quiet in any position when awake, though the spasmodic action was not usually violent. On the first of October, I commenced the employment of electricity, and continued it with but little success for twelve days. A marked amendment then ensued, which was maintained until the 18th, when she was dismissed from my charge entirely recovered.

*Deafness.*—Mrs. ——, in the fall of 1840, had an attack of bilious intermittent fever, recovery from which was followed with deafness in both ears. December 23d, 1842, she called on me for a trial of electricity. I made a direct application to the auditory nerves, twice a week for three months, which gradually restored the organs to their healthy state. This continued until September, 1843, when, from another attack of fever she again became deaf. She has been for a short time past under electrical treatment, and her gradual improvement now indicates an entire recovery in a few weeks.

*Dysmenorrhea.*—Miss ——, aged 30, has suffered from painful menstruation for several years; complains of an almost constant dull aching pain in the small of her back. June 3d, 1843, for the relief of this, made six applications ineffectually. Upon more particular enquiry, found she was subject to habitual constipation. To remedy this, prescribed a mixture of tinct. sem. colchici, 2 ounces; tinct. apocyni, 4 ounces; a teaspoonful to be taken three times a day, alternating every three days, and discontinued the electricity. The daily use of this was, however, resumed on the 28th, and on the 1st of June the menses appeared without dysmenorrheal affection.

The further application of the remedy was omitted, nor has there occurred any occasion for its use since.

*Paralysis.*—Mr. ——, a painter by trade, from the effects of white-lead used in his occupation, had lost the use of both arms, or rather he had what is termed *dropped arms.* If any thing was placed in his hands, he could grasp it apparently as firmly as ever, but had no further control over them. This situation had continued for several years. June 22d, 1843, I commenced applying to the affected limbs continuous and powerful shocks, twice per day, for about ten minutes each application. The paralysis was gradually removed, and on the 14th day he was discharged from my care entirely restored.

